# Major trauma in older persons

**DOI:** 10.1002/bjs5.80

**Published:** 2018-06-23

**Authors:** B. Beck, P. Cameron, J. Lowthian, M. Fitzgerald, R. Judson, B. J Gabbe

**Affiliations:** ^1^ Department of Epidemiology and Preventive Medicine, Monash University Melbourne, Victoria Australia; ^2^ Emergency and Trauma Centre The Alfred Melbourne, Victoria Australia; ^3^ Trauma Service The Alfred Melbourne, Victoria Australia; ^4^ Bolton Clarke Research Institute, Bolton Clarke Melbourne, Victoria Australia; ^5^ National Trauma Research Institute, Royal Melbourne Hospital Melbourne, Victoria Australia; ^6^ Department of General Surgery, Royal Melbourne Hospital Melbourne, Victoria Australia; ^7^ Department of Surgery University of Melbourne Melbourne, Victoria Australia; ^8^ Farr Institute Swansea University Medical School Swansea UK

## Abstract

**Background:**

Globally, populations are ageing, creating challenges for trauma system design. Despite this, little is known about causes of injury and long‐term outcomes in older injured patients. This study aims to describe temporal trends in the incidence, causes and functional outcomes of major trauma in older adults.

**Methods:**

The population‐based Victorian State Trauma Registry was used to identify patients with major trauma aged 65 years and older with a date of injury between 1 January 2007 and 31 December 2016. Temporal trends in population‐based incidence rates were evaluated. Functional outcome was measured using the Glasgow Outcome Scale – Extended.

**Results:**

There were 9250 older adults with major trauma during the study period. Low falls were the most common mechanism of injury (62·5 per cent), followed by transport‐related events (22·2 per cent) and high falls (9·5 per cent). The number of patients with major trauma aged 65 years and older more than doubled from 2007 to 2016, and the incidence increased by 4·3 per cent per year (incidence rate ratio 1·043, 95 per cent c.i. 1·035 to 1·050; P < 0·001). At 12 months after injury, 41·8 per cent of older adults with major trauma had died, and 52·2 per cent of those who survived to hospital discharge were not living independently.

**Conclusions:**

The number and proportion of older adults with major trauma are increasing rapidly and this will impact on trauma system design. Given the poor long‐term outcomes, there needs to be greater emphasis on ensuring that appropriate interventions are targeted to the right patients and enhanced efforts in primary prevention.

## Introduction

Globally, populations are ageing, creating challenges for health systems^1^. Advances in medical care and improved lifestyle factors have resulted in increased longevity, better health and a more active older population. In Australia, the proportion of people aged 65 years or more is projected to grow from the current 15 per cent (3·5 million people) to 21 per cent (8·4 million people) by 2054^2^. This proportional growth in older persons has often been termed the ‘silver tsunami’^3^.

Major trauma has typically been considered a disease of the young, but the mean age of patients with major trauma has increased over time, creating challenges for trauma system design^4^. Data from the UK Trauma Audit Research Network (TARN) demonstrated that the proportion of patients with major trauma aged 75 years and older increased from 8·1 per cent in 1990 to 26·9 per cent in 2013^4^. Compared with younger adults with major trauma, older patients have higher mortality rates, longer hospital and ICU stays, and are more commonly discharged to a nursing home^5^ ^6^. It is thought that these poorer outcomes relate to age‐related physiological changes, and higher rates of pre‐existing co‐morbidity and complications^5^ ^7^.

Previous studies have commonly focused on mortality following major trauma in older persons^6^ ^8^, ^9^ and, as demonstrated in a recent systematic review^10^, there is limited information on the long‐term outcomes in older injured patients. The aims of this study were to describe temporal trends in the incidence, causes and functional outcomes of major trauma in older adults in Victoria, Australia.

## Methods

### Study design

Patients with major trauma, with a date of injury between 1 January 2007 and 31 December 2016, were identified from the Victorian State Trauma Registry (VSTR). Older persons were defined as those aged 65 years or more, consistent with previous research^2^ ^11^, ^12^.

### Setting

The state of Victoria, Australia, has a population of 6·2 million people^13^. The Victorian State Trauma System is an inclusive, organized trauma system that was implemented between 2000 and 2003^14^, with three hospitals (2 adult, 1 paediatric) designated as major trauma services. A single ambulance service provides road and air (fixed‐wing and helicopter) transport of patients.

### Victorian State Trauma Registry

The population‐based VSTR collects data on all patients hospitalized with major trauma in Victoria^8^. A patient is included in the VSTR if any of the following criteria are met: death due to injury; an Injury Severity Score above 12 (Abbreviated Injury Scale, 2008 update); admission to an ICU for more than 24 h; and urgent surgery^4^. The registry collects prehospital and acute care data. Information of longer‐term functional outcome and health status for all survivors to hospital discharge is collected via telephone interview at 6, 12 and 24 months after injury^5^ ^8^. The VSTR has ethics approval from the Department of Health and Human Services Human Research Ethics Committee (HREC) (DHHREC 11/14), the Monash University HREC (CF13/3040 – 2001000165) and 138 trauma‐receiving hospitals in Victoria. Ethics approval for the present study was received from the Monash University HREC.

### Functional outcome

Functional outcome was measured using the Glasgow Outcome Scale – Extended (GOS‐E). The GOS‐E classifies patients into eight levels of function^15^: a GOS‐E score of 1 indicates death, 2 indicates a vegetative state, 3 or 4 indicates lower or upper severe disability, 5 or 6 indicates lower or upper moderate disability, and 7 or 8 indicates lower or upper good recovery. GOS‐E scoring takes into consideration self‐care, activities of daily living, community participation, social and leisure activities, relationships, cognition and work. The GOS‐E can be administered by proxy (for instance by next of kin or carer where direct contact with the patient is not possible) and is recommended for measuring long‐term outcomes of patients with major trauma^16^ ^17^.

### Statistical analysis

Postcodes of residence were mapped to the Accessibility/Remoteness Index of Australia (a geographical index of remoteness) and the Index of Relative Socioeconomic Advantage and Disadvantage (which ranks areas in Australia according to relative socioeconomic advantage and disadvantage). Patients' co‐morbid status was defined using the Charlson Co‐morbidity Index (CCI), mapped from ICD‐10‐AM codes^18^, ^19^, ^20^, with a CCI of zero representing no CCI condition.

Population‐based incidence rates, and 95 per cent confidence intervals, were calculated for each year based on the total population on 30 June for the years 2007–2016. Population estimates for Victoria for each year were obtained from the Australian Bureau of Statistics^13^. Individual Poisson regression models were used to determine whether the incidence rate increased or decreased over the 10‐year period for all older adults with major trauma and for each age group. Data were checked for potential overdispersion (variance greater than the mean) to ensure that the assumptions of a Poisson distribution were met. Incidence rate ratios (IRRs) and 95 per cent confidence intervals were calculated. Patients who sustained injury outside Victoria and were subsequently transported to Victorian hospitals were excluded from incidence calculations.

For specific subanalyses, the GOS‐E at 12 months after injury was dichotomized as lower moderate disability or better (GOS‐E score 5 or more, termed ‘independent living’) or upper severe disability or worse (GOS‐E score of 4 or less). In‐hospital deaths were coded as a GOS‐E score of 1 (death). Patient age was categorized as 65–74, 75–84 and 85 years or more. Comparisons between age groups were made using the χ^2^ test or the Mann–Whitney *U* test, as appropriate, using Stata® version 14.2 (StataCorp, College Station, Texas, USA). *P* < 0·050 was considered significant.

## Results

Over the 10‐year study period, 9250 cases of major trauma in older adults were recorded. These patients were mostly men (55·6 per cent), in the highest quintile for socioeconomic advantage (31·6 per cent), lived in major cities (77·6 per cent), and had injuries resulting from unintentional events (97·7 per cent), most of which occurred in the home (47·8 per cent) (*Table* 1). Low falls were the most common mechanism of injury (62·5 per cent), followed by transport‐related events (22·2 per cent) and high falls (9·5 per cent). Of major injury resulting from low falls, 59·7 per cent occurred in the home, 19·7 per cent in residential institutions, 7·0 per cent on the street, 2·8 per cent while in hospital and 10·8 per cent in other locations.

**Table 1:** Demographic, event and injury characteristics for older adults with major trauma

	Overall (*n* = 9250)	Age 65–74 years (*n* = 2829)	Age 75–84 years (*n* = 3549)	Age ≥ 85 years (*n* = 2872)	*P* ¶
Sex					< 0·001
M	5144 (55·6)	1948 (68·9)	1959 (55·2)	1237 (43·1)	
F	4106 (44·4)	881 (31·1)	1590 (44·8)	1635 (56·9)	
Charlson Co‐morbidity Index					0·003
0	4942 (53·4)	1562 (55·2)	1818 (51·2)	1562 (54·4)	
≥ 1	4308 (46·6)	1267 (44·8)	1731 (48·8)	1310 (45·6)	
IRSAD (quintiles)†					< 0·001
1st (most disadvantaged)	1313 (14·5)	442 (16·1)	528 (15·2)	343 (12·1)	
2nd	1313 (14·5)	443 (16·1)	503 (14·5)	367 (13·0)	
3rd	1617 (17·9)	535 (19·4)	610 (17·6)	472 (16·7)	
4th	1946 (21·5)	567 (20·6)	789 (22·7)	590 (20·9)	
5th (least disadvantaged)	2863 (31·6)	766 (27·8)	1044 (30·1)	1053 (37·3)	
ARIA‡					< 0·001
Major cities of Australia	7018 (77·6)	1923 (70·0)	2724 (78·5)	2371 (84·0)	
Inner regional/outer regional/remote Australia	2024 (22·4)	823 (30·0)	748 (21·5)	453 (16·0)	
Event type					< 0·001
Unintentional event	9033 (97·7)	2709 (95·8)	3484 (98·2)	2840 (98·9)	
Intentional, self‐harm	77 (0·8)	37 (1·3)	27 (0·8)	13 (0·5)	
Intentional, other	94 (1·0)	64 (2·3)	23 (0·6)	7 (0·2)	
Not determined	46 (0·5)	19 (0·7)	15 (0·4)	12 (0·4)	
Mechanism of injury					< 0·001
Transport‐related	2055 (22·2)	924 (32·7)	797 (22·5)	334 (11·6)	
Low fall (≤ 1 m)	5779 (62·5)	1088 (38·5)	2288 (64·5)	2403 (83·7)	
High fall (> 1 m)	876 (9·5)	510 (18·0)	291 (8·2)	75 (2·6)	
Other	540 (5·8)	307 (10·9)	173 (4·9)	60 (2·1)	
Location of injury					< 0·001
Home	4417 (47·8)	1221 (43·2)	1792 (50·5)	1404 (48·9)	
Residential institution	1172 (12·7)	92 (3·3)	342 (9·6)	738 (25·7)	
Road, street or highway	2336 (25·3)	965 (34·1)	931 (26·2)	440 (15·3)	
Other	1325 (14·3)	551 (19·5)	484 (13·6)	290 (10·1)	
Definitive care					< 0·001
Major trauma service	6542 (70·7)	2294 (81·1)	2565 (72·3)	1683 (58·6)	
Other	2708 (29·3)	535 (18·9)	984 (27·7)	1189 (41·4)	
Injury Severity Score*, §	17 (13–25)	17 (14–25)	17 (13–25)	17 (13–25)	< 0·001#

Values in parentheses are percentages unless indicate otherwise;

*values are median (i.q.r.).

Data missing for †198, ‡208 and §ten patients.

IRSAD, Index of Relative Socioeconomic Advantage and Disadvantage;

ARIA, Accessibility/Remoteness Index of Australia.

¶χ^2^ test, except #Mann–Whitney *U* test.

With increasing age, the proportion of injured women increased, the proportion of events resulting from transport events and high falls decreased, and the proportion of events resulting from low falls increased (*Table* 1). Women represented 22·6 per cent of patients with major trauma at age 65 years, increasing to 65·5 per cent at 95 years of age (*Fig*. S1, supporting information). The proportion of patients who had definitive care at a major trauma service was 81·1 per cent in 65–74‐year‐olds, 72·3 per cent in 75–84‐year‐olds and 58·6 per cent in those aged 85 years or above (*Table* 1).

### Temporal trends

**Figure 1:**
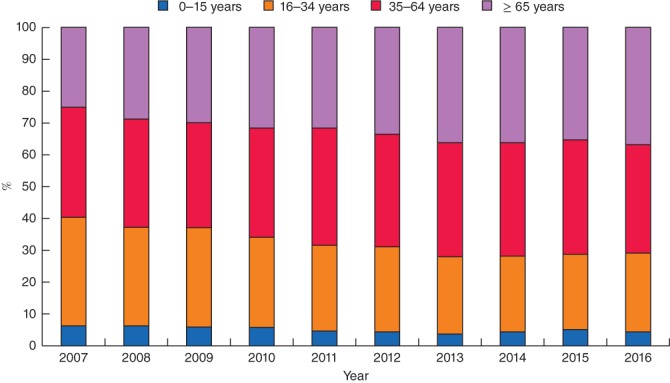
Changes in the distribution of major trauma by age group between 2007 and 2016

**Figure 2:**
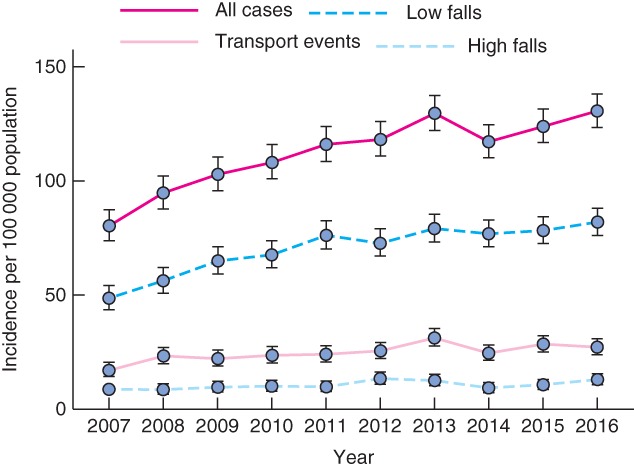
Changes in the incidence of major trauma in older adults, overall and by mechanism of injury, between 2007 and 2016. Values are incidence rates and 95 per cent confidence intervals

**Figure 3:**
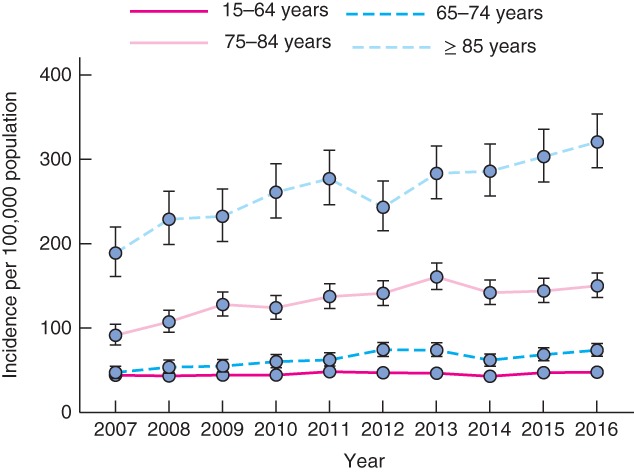
Changes in the incidence of major trauma by age group between 2007 and 2016. Values are incidence rates and 95 per cent confidence intervals

The proportion of patients with major trauma who were aged 65 years or more increased from 25·1 per cent in 2007 to 36·7 per cent in 2016 (*Fig*. 1). The number of older adults with major injury increased from 572 in 2007 to 1217 in 2016. The incidence of major trauma in older adults increased by 4·3 per cent per year (IRR 1·043, 95 per cent c.i. 1·035 to 1·050; *P* < 0·001) (*Fig*. 2). This rate was greater than in those aged 15–64 years, in whom the incidence increased by 0·8 per cent per year (IRR 1·008, 1·003 to 1·013; *P* = 0·004). The incidence of major trauma increased in all older age groups: 4·1 per cent per year in those aged 65–74 years (IRR 1·041, 1·027 to 1·055; *P* < 0·001), 4·4 per cent per year in those aged 75–84 years (IRR 1·044, 1·032 to 1·057; *P* < 0·001) and 4·7 per cent in those aged 85 years or more (IRR 1·047, 1·033 to 1·061; *P* < 0·001) (*Fig*. 3).

Increases in the incidence of major trauma in older adults were observed for low falls (IRR 1·047, 95 per cent c.i. 1·037 to 1·056; P < 0·001), high falls (IRR 1·035, 1·011 to 1·060; P = 0·004) and transport events (IRR 1·040, 1·024 to 1·056; P < 0·001) (Fig. 2).

### Outcomes following major trauma

The in‐hospital mortality rate for older adults with major injury was 25·6 per cent, rising from 13·2 per cent in people aged 65–74 years to 39·2 per cent in those aged 85 years and older (Table 2). Most patients who survived to discharge were discharged to a rehabilitation facility (53·7 per cent) or home (30·5 per cent) (Table 2). A greater proportion of patients aged 65–74 years were discharged home compared with older age groups.

**Table 2:** Hospital outcomes following major trauma in older adults

	Overall (*n* = 9250)	Age 65–74 years (*n* = 2829)	Age 75–84 years (*n* = 3549)	Age ≥ 85 years (*n* = 2872)	*P* ‡
ICU stay					< 0·001
No	6473 (70·0)	1681 (59·4)	2350 (66·2)	2442 (85·0)	
Yes	2777 (30·0)	1148 (40·6)	1199 (33·8)	430 (15·0)	
Duration of hospital stay (days)*, †	7·6 (3·9–13·8)	8·0 (4·3–14·8)	7·9 (4·1–14·6)	6·6 (3·2–12·1)	< 0·001§
In‐hospital mortality					< 0·001
No	6880 (74·4)	2456 (86·8)	2677 (75·4)	1747 (60·8)	
Yes	2370 (25·6)	373 (13·2)	872 (24·6)	1125 (39·2)	
Discharge destination (for those surviving to hospital discharge)					< 0·001
Home	2099 (30·5)	1004 (40·9)	708 (26·4)	387 (22·2)	
Rehabilitation	3696 (53·7)	1227 (50·0)	1534 (57·3)	935 (53·5)	
Nursing home	295 (4·3)	19 (0·8)	111 (4·1)	165 (9·4)	
Hospital for convalescence	631 (9·2)	173 (7·0)	263 (9·8)	195 (11·2)	
Other	159 (2·3)	33 (1·3)	61 (2·3)	65 (3·7)	

Values in parentheses are percentages unless indicate otherwise;

* values are median (i.q.r.).

† Data missing for six patients.

‡ χ^2^ test, except

§ Mann–Whitney *U* test.

**Figure 4:**
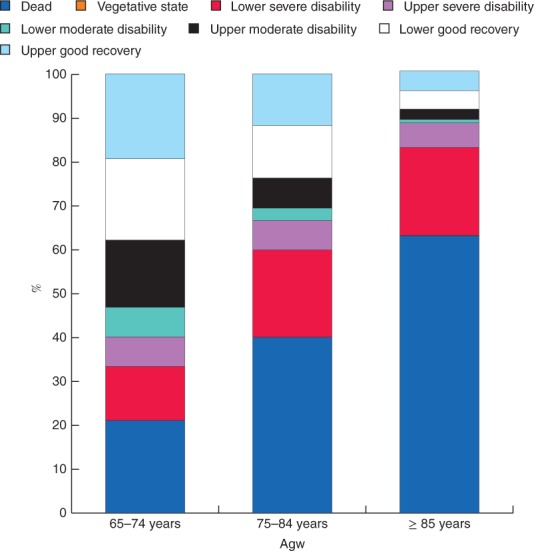
Glasgow Outcome Scale – Extended (GOS‐E) at 12 months after injury for patients with major trauma aged 65–74, 75–84 and 85 years or more. In‐hospital deaths were included in this analysis and coded as GOS‐E score 1

At 12 months after injury, 87·8 per cent of older adults with major trauma had completed a valid GOS‐E. Compared with those followed up, patients lost to follow‐up had a lower proportion of unintentional events, a greater proportion of events resulting from transport crashes and high falls, and a greater proportion of events that occurred in the home (Table S1, supporting information). Of patients with a valid GOS‐E completed at 12 months after injury (including in‐hospital deaths), 34·2 per cent were classified as living independently and 41·8 per cent had died (Table 3). Of those who survived to hospital discharge, 47·8 per cent were living independently at 12 months after injury. There were clear age‐related differences in functional outcomes at 12 months after injury (Table 3 and Fig. 4). The proportion of patients who had a lower or upper good recovery (GOS‐E score 7 or 8) decreased from 37·7 per cent in people aged 65–74 years to 23·7 per cent in those aged 75–84 years and 8·6 per cent in those aged 85 years or more.

**Table 3:** Functional outcomes measured using the Glasgow Outcome Scale – Extended 12 months after injury

	Overall (*n* = 8128)	Age 65–74 years (*n* = 2408)	Age 75–84 years (*n* = 3126)	Age ≥ 85 years (*n* = 2594)	*P* †
GOS‐E at 12 months after injury*					< 0·001
Dead	3394 (41·8)	510 (21·2)	1253 (40·1)	1631 (62·9)	
Vegetative state	8 (0·1)	2 (0·1)	4 (0·1)	2 (0·1)	
Lower severe disability	1433 (17·6)	295 (12·3)	620 (19·8)	518 (20·0)	
Upper severe disability	513 (6·3)	163 (6·8)	211 (6·7)	139 (5·4)	
Lower moderate disability	262 (3·2)	157 (6·5)	82 (2·6)	23 (0·9)	
Upper moderate disability	646 (7·9)	372 (15·4)	215 (6·9)	59 (2·3)	
Lower good recovery	938 (11·5)	446 (18·5)	381 (12·2)	111 (4·3)	
Upper good recovery	934 (11·5)	463 (19·2)	360 (11·5)	111 (4·3)	

Values in parentheses are percentages.

* Data missing (lost to follow‐up) for 1122 patients. In‐hospital deaths were included in the analysis and coded as Glasgow Outcome Scale – Extended (GOS‐E) score 1.

† χ^2^ test.

**Figure 5:**
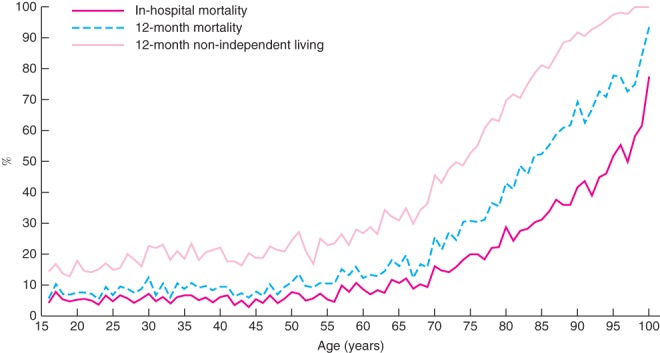
In‐hospital mortality, 12‐month mortality and 12‐month functional outcomes after major trauma according to age. Non‐independent living was used for functional outcome, defined as a Glasgow Outcome Scale – Extended score of 4 or less. Values shown at 100 years include all patients with major injury aged 100 years or more

The rates of in‐hospital mortality, 12‐month mortality and 12‐month functional outcomes were relatively stable in patients aged 16–64 years (*Fig*. 5). A sharp and consistent increase in poor outcomes was observed in patients older than 65 years. Of patients aged 85 years, 52·5 per cent had died at 12 months after injury and 81·2 per cent were not living independently (*Fig*. 5). At age 90 years, 69·3 per cent had died at 12 months after injury and 91·7 per cent were not living independently.

## Discussion

This study investigated the epidemiology and long‐term outcomes of major trauma in older persons over a 10‐year period. The absolute number of such patients more than doubled, and there was an increase in the proportion of patients with major trauma aged 65 years and older, from 25·1 per cent in 2007 to 36·7 per cent in 2016. At 12 months after injury, 41·8 per cent of older adults with major trauma had died and, of those who survived to hospital discharge, 52·2 per cent were not living independently.

These findings of a shift in the age profile of major trauma are consistent with changes observed in the UK. Kehoe and colleagues^4^ demonstrated an increase in the proportion of patients with major trauma aged 75 years or above, from 8·1 per cent in 1990 to 26·9 per cent in 2013. Similarly, Dinh and colleagues^11^ observed an increase in the proportion of patients with major injury aged 65 years and older, from 20 per cent in 1991 to 33 per cent in 2010, at a single trauma centre in Australia. Although these previous studies did not account for population changes, the present authors have demonstrated that these increases far outweigh population growth.

There are numerous possible explanations for the observed increase in the incidence of major trauma in older persons. CT is increasingly being used in the primary assessment of injured patients. In the UK, the proportion of patients with major trauma who underwent CT increased from 34 per cent in 1990 to 87 per cent in 2013^4^. The increased use of CT may have led to greater detection of injuries that may have been missed previously, and therefore to an increase in the proportion of patients classified as having major injury. In addition, the initial referral, assessment and expectations of treatment for older persons have changed. Falls in residential care and the community in frail older persons are now routinely assessed with CT, independent of the likely clinical intervention and benefit. Further research is required to understand whether a proportion of the observed increases in older adult major trauma are linked to this improved injury detection.

Few previous studies have described long‐term outcomes following trauma in older adults^10^. Of these, most focused on hip fracture or traumatic brain injury^10^. In addition to high rates of in‐hospital mortality, the present study demonstrated that 41·8 per cent of the older adult major trauma population had died and 65·8 per cent had an unfavourable outcome at 12 months after injury. Gabbe *et al*.^21^ previously showed that functional outcomes decline the longer the time after injury, particularly in those aged 75 years or more.

Given the shift in major trauma to a greater proportion of older adults, it must be ensured that trauma systems are adapted to these changes. It is known^22^, ^23^, ^24^ that the physiological response to trauma in older adults is different from that in younger adults, and prehospital triage tools need to reflect this. In the present study, as the age of the patient increased there was a decrease in the likelihood of the patient being managed at a major trauma service. This may be explained by patients being critically unwell and/or dying before interhospital transfer, but there may also be a degree of undertriage in older persons – a factor that has been observed in other settings^12^. It is essential for trauma clinicians to understand the predicted outcomes for patients with major injury following acute hospital admission, in order to target appropriate interventions and include family members in individualized treatment plans. Given the poor outcomes observed in older patients with major trauma, particularly those aged 85 years or more, aggressive interventions to ensure survival may not be appropriate for some. In patients with significant frailty and co‐morbidity, the injury may represent the final pathway to death or severe disability.

Given the complex ongoing healthcare needs of older injured patients, particularly in the setting of cognitive impairment and polypharmacy, it has been suggested^25^ that a comprehensive geriatric assessment may result in better outcomes. Furthermore, older patients managed at trauma centres that treat a higher proportion of older people with injury have been shown to have lower in‐hospital mortality rates^26^. As a result, it has been proposed that treating these patients in trauma centres that specialize in older adult trauma may improve outcomes. Regardless, prehospital, in‐hospital and postdischarge care must evolve to better meet the needs of older persons following trauma. This may include conjoint management under medical and geriatric units.

Consistent with previous studies^4^ ^11^, ^26^, this study has demonstrated that low falls are the most common mechanism of injury in older adults with major trauma. Low falls are now a major public health issue and are the leading cause of injury hospitalization in Australia^27^. Although advances in prehospital and in‐hospital management of older adults with major injury may improve outcomes, the most effective method to curb the rising incidence is clearly through primary prevention activities. Interventions shown to reduce falls include group and home‐based exercise programmes, home safety interventions and improved home design, and reductions in polypharmacy^28^. High falls were also observed in the present study as a common cause of major injury in older adults; these commonly result from falls from ladders^29^ ^30^. It is clear that effective interventions at a community and population level are warranted, and it has been suggested^29^ that helmets may be required for ladder users in domestic settings.

This study is not without limitations. It focused on older patients hospitalized with major trauma and did not include prehospital deaths; there may be temporal changes in the prehospital prognostication of older adults with major trauma that were not accounted for. Furthermore, and as discussed above, some of the increases in the incidence of older adult major trauma may be explained by increased use of CT and changes in community expectations regarding the management of acute illness in the older population. This has been raised as an issue in previous studies^4^ and was a factor that it was not possible to account for. Additionally, limitations of treatment orders for patients in residential and supported accommodation were not considered.

The number and proportion of identified major trauma cases in older persons are increasing rapidly and this will impact on trauma system configuration, with implications for trauma triage, reception and trauma service delivery. Given the often poor in‐hospital and long‐term outcomes following major trauma in older adults, there is a need to develop accurate prediction models and include patient treatment preferences in decision‐making to ensure that appropriate interventions are targeted to the right patients. There are also significant opportunities to improve primary prevention activities to reduce the burden of injury in older adults.

## Supporting information


**Fig. S1** Proportion of male and female patients with major trauma for each year of age. The dot representing age 100 years reflects all patients with major trauma aged 100 years or more
**Table S1** Comparison of patients followed up at 12 months after major injury and those lost to follow‐up
