# Supplementary material for: Major trauma in older persons
Source: BJS Open. 2018 Jun 23;2(5):310–8. doi: 10.1002/bjs5.80 (PMC6156159; doi:10.1002/bjs5.80)
Supplement: Supplementary file 1 — Fig. S1 Proportion of male and female patients with major trauma for each year of age. The dot representing age 100 years reflects all patients with major trauma aged 100 years or more Table S1 Comparison of patients followed up at 12 months after major injury and those lost to follow‐up [file BJS5-2-310-s001.docx]

**BJS5_80**

**Major trauma in older persons**

**B. Beck, P. Cameron, J. Lowthian, M. Fitzgerald, R. Judson and B. J Gabbe**

**Fig. 1** Proportion of male and female patients with major trauma for each year of age. The dot representing age 100 years reflects all patients with major trauma aged 100 years or more

**Table S1** Comparison of patients followed up at 12 months after major injury and those lost to follow-up

|  | **Followed up** | **Lost to follow-up** | **P-value** |
| --- | --- | --- | --- |
| N | 8,128  (87.9%) | 1,122  (12.1%) |  |
| *Sex* |  |  | 0.338 |
| Male | 4,535  (55.8%) | 609  (54.3%) |  |
| Female | 3,593  (44.2%) | 513  (45.7%) |  |
| *Charlson Comorbidity Index* |  |  | 0.090 |
| 0 | 4,316  (53.1%) | 626  (55.8%) |  |
| ≥1 | 3,812  (46.9%) | 496 (44.2%) |  |
| *IRSAD (quintiles) ^a^* |  |  | 0.145 |
| 1^st^ (most disadvantaged) | 1,137  (14.3%) | 176  (16.2%) |  |
| 2^nd^ | 1,156  (14.5%) | 157  (14.4%) |  |
| 3^rd^ | 1,422  (17.9%) | 195  (17.9%) |  |
| 4^th^ | 1,741  (21.9%) | 205  (18.8%) |  |
| 5^th^ (least disadvantaged) | 2,508  (31.5%) | 355  (32.6%) |  |
| *ARIA ^b^* |  |  | 0.980 |
| Major cities of Australia | 6,174  (77.6%) | 844  (77.6%) |  |
| Inner regional / outer regional / remote Australia | 1,781  (22.4%) | 243  (22.4%) |  |
| *Fund ^c^* |  |  | 0.754 |
| Non-compensable | 6,436  (79.9%) | 883  (79.5%) |  |
| Compensable | 1,621  (20.1%) | 228  (20.5%) |  |
| *Event type* |  |  | 0.039 |
| Unintentional event | 7,949  (97.8%) | 1,084  (96.6%) |  |
| Intentional-self harm | 67  (0.8%) | 10  (0.9%) |  |
| Intentional-other | 75  (0.9%) | 19  (1.7%) |  |
| Not determined | 37  (0.5%) | 9  (0.8%) |  |
| *Mechanism of Injury* |  |  | 0.042 |
| Transport-related | 1,792  (22.1%) | 263  (23.4%) |  |
| Low fall (≤1 m) | 5,117  (63.0%) | 662  (59.0%) |  |
| High fall (>1 m) | 759  (9.3%) | 117  (10.4%) |  |
| Other | 460  (5.7%) | 80  (7.1%) |  |
| *Location of injury* |  |  | <0.001 |
| Home | 3,846  (47.3%) | 571  (50.9%) |  |
| Residential institution | 1,097  (13.5%) | 75  (6.7%) |  |
| Road, street or highway | 2,031  (25.0%) | 305  (27.2%) |  |
| Other | 1,154  (14.2%) | 171  (15.2%) |  |
